# The Risk Factor of Age in Construction Accidents: Important at Present and Fundamental in the Future

**DOI:** 10.1155/2018/2451313

**Published:** 2018-12-24

**Authors:** Miguel A. Camino López, Oscar J. González Alcántara, Ignacio Fontaneda, Mar Mañanes

**Affiliations:** ^1^Department of Civil Engineering, University of Burgos, c/Villadiego s/n, 09001 Burgos, Spain; ^2^Department of Civil Engineering, University of Burgos, Avda. Cantabria s/n, 09006 Burgos, Spain

## Abstract

Occupational accidents in the construction sector are analyzed in this study of the relation between the age of the injured worker, days off work due to the injury, and accident severity. A further aim is to establish whether accumulated fatigue during the working day and throughout the week has a similar effect on all workers, regardless of age. A total of three million four hundred and thirty-eight thousand, one hundred and forty-five (3,438,145) accidents are analyzed in this study; the total of all accidents are notified in Spain by workers from the sector over the period 1996-2015. The results confirmed a direct link between worker age and both days off work due to the injury and accident severity. They also confirmed that the workers injured in accidents in the afternoon hours were older than the workers injured in the morning hours. In consequence, the average number of days off work due to injury following an accident of the older workers was also larger and the accidents are more severe. Likewise, the workers injured on a Friday were of an older average age than those injured on a Monday. In consequence, the average number of days off work due to injury on the last day of the week was also higher and the accidents were of more severity. All the above-mentioned differences were statistically significant.

## 1. Introduction

Over recent years, it is becoming evident that the average age of the workforce in a large part of the European Union is increasing, due to population aging and the prolongation of the active working life of people. In fact, while the average working age was 30 years in 1980, it rose to 41 years in 2013 [1], and it is expected that workers of 55 to 64 years old will represent 30% or more of the active population in many countries [2]. Studies on the subject in the USA inform us that the advanced age of the workforce in the construction industry has no influence on the number of injuries; however, age is related to a higher cost of injuries. That higher cost is probably due, in part, to the severity of the injuries inflicted on older workers [3].

Different authors have investigated age as a risk factor in accident rates, arriving at different conclusions by country and by activity. In fact, Salminen [4] classified the results of an international review of articles in accordance with whether younger workers suffered more injuries than older workers. It was verified from the results of a review of 63 papers on nonfatal accidents that higher incidence rates were associated with young people in 56% of the papers; no differences were found in 27%, and it was considered lower in 17%. Severity was also analyzed in a review of 45 papers on fatal accidents; to conclude that the ratio of young people was considered higher in 16%, no differences were noted in 20%, and it was considered very low in 64%.

Other studies have analyzed the incidence of age on both fatal and nonfatal falls. Thus, in a study on accident rates in the USA due to falls to the same level, over the period 2006-2010, it was concluded that the accident rate ratio increased as the age of the worker advanced. In 2010, the ratio of falls to the same level of workers aged 20-24 years was in fact 10.7, while workers older than 65 registered 34.6 of falls. However, the youngest registered 16.2 of falls. In addition, those falls recorded a very high number of days off, because over 29% of falls caused 31 or more days off work [5].

Some authors have sought to show the influence of age and other factors on the duration of accident-related sick leave. Thus, the days off work among the Belgium working population were studied over the period 2001-2003, finding that, except for the group of 60-69 years old, the time off work increased as their age increased. Likewise, a lengthier duration of sick leave was associated with construction activity, blue collar workers, and a smaller size of firm [6].

Centering on the construction sector, the authors of various studies have attempted to relate age with other variables and circumstances. Thus, it was found that the average age of workers injured in construction work in the USA was 37.2 years, as against those registered for accidents due to falls that amounted to 38.3 in 1990-2001 [7]. With regard to fatal falls involving construction workers, it was found that the youngest workers suffered lower percentages of falls than the oldest workers. However, after introducing a new risk factor, ethnic origin, they found that Hispanic workers aged between 16 and 24 registered 18.3% of all fatal falls, while non-Hispanic white workers of that same age range suffered 6.5% and in all construction work, 10.1% [8].

In another study, Chau et al. [9] concluded that ages of over 30 years were correlated with hospitalization and lengthier periods of sick leave. Differences were also observed in relation to the injury. Thus, following the analysis of 136,000 injuries to construction workers, it was found that workers over 35 years old had a higher risk of injuries to their knees and shoulders, while the cuts and wounds of those under 35 years stood out [10]. Older workers were found not to be at increased risk of a fall injury but were more likely to be hospitalized after injury than younger workers [11].

In other words, the older age of workers can have consequences for the accident rates in all productive sectors and, particularly, in the construction sector. Therefore, we consider it necessary to deepen the investigation into the influence that the advanced age of the workforce is having and will have in the future on the occupational accident rates of a country.

With regard to working hours and fatigue, other authors have sought to demonstrate the relation between fatigue and errors at work, in some cases going so far as to demonstrate the existence of a linear relation between those two variables [12].

In relation to the hour of the accident and the accumulation of fatigue throughout the day, the highest accident-related ratios in the construction industry of Oregon were found to occur in the 3rd and the 4th hour of the working day [13]. Moreover, in another study, Goldenhar [14] reported that a number of workers in his study established a direct relation between working overtime and their personal experiences with occupational injuries. Other authors studied the hour of the accident as a partial aspect of their investigations into accidents due to certain causes. Thus, when analyzing fatal falls in the construction industry of the United States of America, it was determined that 76% took place between 6:00 am and 5:00 pm [15]. Analyzing nonfatal falls in the state of Virginia, it was found that 85% of all injuries due to falls took place between 8:00 am and 4:00 pm [16]. With regard to blows, the most frequent times were 9:00 and 13:00 hours [17]. A greater number of injuries and deaths from electric shocks occurred occurring at 10:00 am, were less frequent at lunch time, and then increased spectacularly, only to diminish in the late afternoon [18].

This question has also been studied in other countries. Thus, in Portugal, the time band with the highest accident rates was found to be between 8:00 to 12:00 hours [19]. Likewise, it was found that most injuries in Australia were inflicted in the morning hours rather than in the afternoon on all working days of the week [20]. In both Sweden and Denmark, an analysis of falls to another level involving construction workers concluded that the highest number of falls took place before 13:00 hours. However, surprisingly, the highest number of fatal falls took place in the afternoon [21].

However, the relation that may exist between the hour of the day and the age of the injured worker has yet to be investigated. In this study, we shall investigate the possible influence of the age of the worker on the accumulated fatigue both during the working day and throughout the week, i.e., normal accumulation of fatigue from Monday to Friday. We hypothesize that the accumulation of fatigue, during both the working day and the working week, will have negative repercussions to a greater extent on older workers than on younger workers.

In addition, three variables will be analyzed of evident importance in accident rates in the construction sector: the age of the injured worker, days off work due to injury, and the severity of the accident. Their evolution and the relation between them will be studied, through an analysis of the accidents in the Spanish construction sector over the period 1996-2015.

## 2. Materials and Methods

### 2.1. Data Collection

All occupational accidents in Spain that, as a consequence of injury, involve an absence from work equal to or more than one day must be reported to the Labor Authority (MEySS, Ministerio de Empleo y Seguridad Social). In addition, taking into account the fact that the daily sick benefit paid out to a worker is notably higher when the accident is ascribed to work, we can assume that almost 100% of all accidents are notified to the authorities in Spain.

We can therefore be certain that practically all accidents in Spain that involve salaried constructions workers over the period 1996-2015 were included in the study. The number of accidents under analysis amounted to three million four-hundred and thirty-eight thousand, one hundred and forty-five (3,438,145). Relapses from earlier accidents (102,096 cases) were excluded from the study, as well as those accidents, which due to lack of information, specified 0 days of absence from work (6,492 cases), and due to its exceptionality, those involving workers aged over 66 years (2,139 cases), as the ordinary retirement age in Spain, in 2015, was approximately 65 years and three months.

In Spain, medical staff is responsible for the diagnosis of the severity of accidents [22] under subjective criteria. Labor inspection provides the Instruction 104/2001 of the General Directorate of the Labor and Social Security Inspectorate [23] by which the ILO takes into account the application of its penalties [24]; in section 2.3.1.g, it is established that severe injuries will be considered “those which may cause the worker motivational damage that could potentially imply a declaration of permanent disability (total or absolute), or injuries, mutilations, or definite deformities (even in case of not being invalidating), whenever the anatomical or functional losses are severe”. From among the accidents under analysis, three million, three-hundred and eighty-five thousand, two hundred and twenty-eight accidents (98.46%) were registered as minor accidents, while fifty-two thousand, nine hundred and seventeen were registered as severe and fatal (1.54%).

### 2.2. Study Design

Obtaining all the information on the total of all accidents and knowing the severity assigned to each one, we shall attempt to establish the possible relation between the severity of the accidents, the number of days off work due to the injury, and the age of the injured worker.

Thus, in this study, we propose to test the following hypothesis.

**Hypothesis 1 .**

The age of the injured worker will be directly related to the severity of the accident. In other words, an older age will imply greater accident-related severity.

**Hypothesis 2 .**

The age of the worker involved in the accident will be directly related to the duration of the accident-related sick leave. An older age of the worker involved in the accident will imply a longer period of days of work due to the injury.

The accumulated fatigue in a working day and throughout the week will not affect all workers equally, but will affect the older workers with greater intensity. Therefore:

**Hypothesis 3 .**

Workers involved in accidents that occur in the afternoon have a higher average age and, in consequence, the accidents will be of greater severity and will be followed by a higher average number of days off work due to the injury than those registered in accidents in the morning hours

**Hypothesis 4 .**

Workers involved in accidents on Fridays will be associated with those of an older average and, in consequence, will imply a greater severity and will involve higher average numbers of days off work due to the injury than those recorded on Mondays, Tuesdays, Wednesdays, and Thursdays.

### 2.3. Statistical Analysis

Determination of the differences between independent means in the statistical analyses of accident severity, age of the injured worker, and days off work due to the injury was done with the Student t-test. Equality of variance was not assumed, calculating the upper and lower confidence intervals from those differences at a confidence level of 95%.

When it was necessary to compare a discrete and another continuous variable, single-factor ANOVAS were used, calculating the intervals of confidence for the average at a confidence level of 95%.

The percentages of severe accidents in each group were calculated for the treatment of severity as follows: the severe plus the fatal accidents were divided by the total of all accidents and then the result was multiplied by 100.

The Percent Differences method was employed to compare the resulting percentages, by calculating the Z value and the p-value of the intervals with

(1) z=p−1−p−2p−11−p−1/n1+p−21−p−2/n2

(2) I=p−1−p−2±z∝/2·p−11−p−1n1+p−21−p−2n2

The study was also done, considering the distributions of frequency of the severity as a continuous variable; to do so, a value of 0 was assigned to minor accidents and a value of 1 to severe and fatal accidents (S+F). Subsequently, the Student t-test of differences of independent means was completed.

All the analyses were done using the SPSS v23 statistical software package.

## 3. Results and Discussion

In Table 1, the evolution of the Spanish construction sector and its descriptive statistics may be observed over the period 1996-2015.

We may highlight, in the first place, the notable reduction recorded for the total number of accidents, as well as severe and fatal ones. It should be recalled that this decrease is, in the first place, fundamentally due to the significant economic crisis that Spain has and is still weathering since 2008. This crisis has had the most intense impact of all on the construction sector.

Another aspect to highlight is that the number of Severe+Fatal accidents has shown a similar trend to the one registered by minor accidents. However, the same did not happen over the period 2002-2003, when the number of severe and fatal accidents increased while the total number of accidents diminished. We may infer that the number of severe accidents will not always have a direct relation with the number of minor accidents.

**Figure 1:**
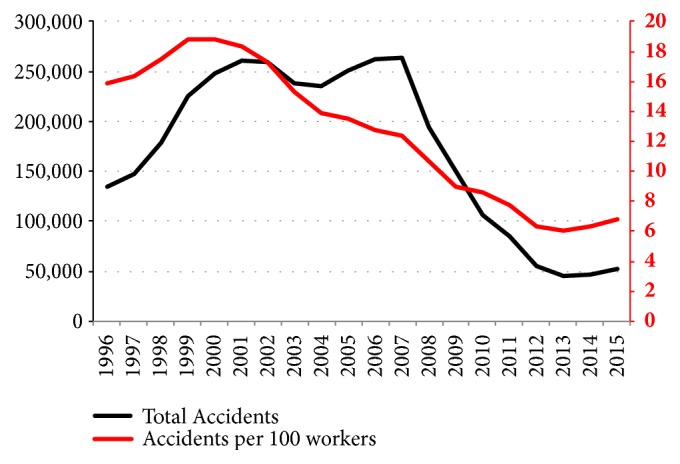
Number of accidents and incidence rate over time. Spain 1996-2015.* Source.* Prepared by the authors using the anonymous data on occupational accidents provided by the Spanish Subdirección General de Estadística of the Ministerio de Empleo y Seguridad Social.

We can see from Figure 1, with its two ordinate axes, that the Incident Rate curve (accidents per 100 workers) does not follow the same curve as the number of accidents. Thus, in the first years of the study, both the number of accidents and the accident rate increased each year. However, as from 2001, a constant reduction took place in rates, notwithstanding the increasing number of accidents. Efforts invested in safety at work appear to have yielded positive results. On a negative note, we can highlight the increase experienced in the incidence rate over the past two years, while noting the need to investigate the causes that provoked such an increase.

In what follows, the results are detailed from the analysis of the variables: age, accident severity, and number of days off work due to the injury, with a view to testing the proposed hypothesis (see Section 2.2).

### 3.1. Age, Days off Work due to the Injury, and Accident Severity

As illustrated by Table 1, the average age of the workers involved in accidents over the period 1996-2015 stood at 35.31 years, passing from 35.27 years in 1996 to 40.73 in 2015. The lowest average age was registered in 2001 (34.28) and the highest, as mentioned above, in 2015.

The average number of days off work due to the injury following accidents involving construction workers over the period 1996-2015 was set at 24.01 days, passing from 25.51 in 1996 to 33.94 in 2015. The lowest number of days off work due to the injury was recorded in 2000 (22.08) and the highest, as mentioned, in 2015.

The severity and the fatality of the accidents have undergone frequent variations over the twenty years under analysis. We can highlight the successive reductions over the period 2004-2009, passing from 1.82% of Severe+Fatal (S+F) accidents in 2003 to 1.24% in 2009. We can conclude by saying that the percentages of severe and fatal accidents have been reduced by almost 27%, passing from 1.97% in 1996 to 1.44% in 2015.

#### 3.1.1. Hypothesis 1: Age and Accident Severity

**Figure 2:**
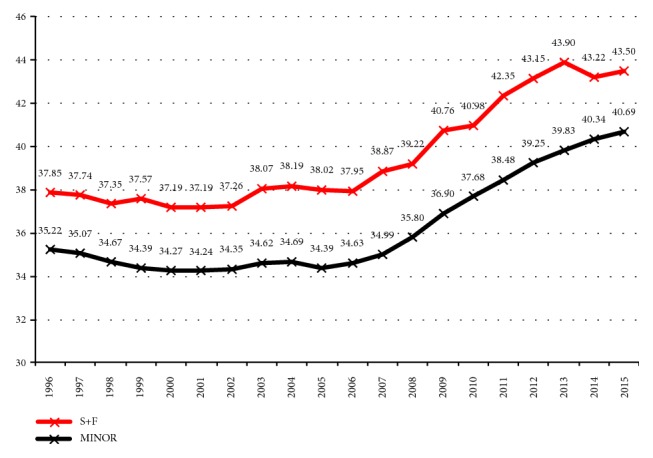
Average ages of workers injured in minor accidents and severe and fatal (S+F) accidents. Spain 1996-2015.* Source.* Prepared by the authors using the anonymous data on occupational accidents provided by the Spanish Subdirección General de Estadística of the Ministerio de Empleo y Seguridad Social.

The average age of workers involved in a minor accident stood at 35.26 and of those involved in a severe or fatal accident at 38.47 (t: -62.212; CL 95% of the difference: lower -3.3071, upper -3.1147). In Figure 2, the average numbers of workers involved in severe or fatal accidents may be seen alongside the average age of workers involved in minor accidents.

We found that the average age of the workers involved in severe-fatal accidents over the twenty years under analysis was higher than those registered by workers involved in minor accidents. That average difference was 3.2 years, registering the highest difference in 2013 (4.1) and the lowest one in 2015 (2.8).

**Figure 3:**
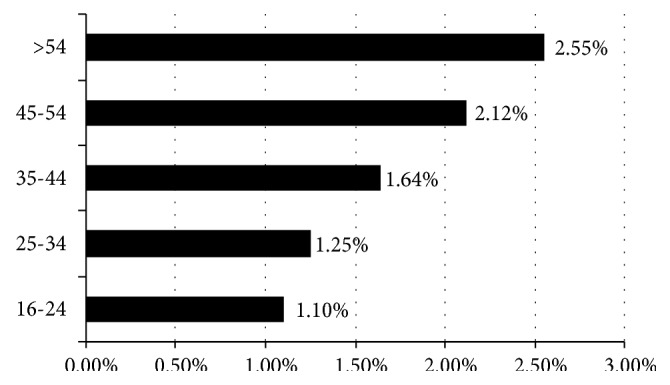
Percentage of severe and fatal accidents by age. Spain 1996-2015.* Source.* Prepared by the authors using the anonymous data on occupational accidents provided by the Spanish Subdirección General de Estadística of the Ministerio de Empleo y Seguridad Social.

Establishing age bands, it may be seen in Figure 3 that, in effect, differences were evident between the percentage of Severe+Fatal accidents inflicted on the youngest workers (group of 16-24 years old) and all other workers. These differences presented statistical significance. Thus, a difference of 0.15% (t: -9.519; CL 95% of the difference: lower -0.19%, upper -0.12%) was registered for the age group of 16-24 years old and the age group of 25-34 years old; a difference of 0.54% (t: -29.202; CL 95% of the difference: lower -0.58%, upper -0.51%) was registered for the age group of 35-44 years old; a difference of 1.12% (t: -43.604; CL 95% of the difference: lower -1.07%, upper -0.98%) for the age group of 45-54 years old; and finally, the difference was 1.45% (t: -40.835; CL 95% of the difference: lower -1.52%, upper -1.38%) for the oldest age group.

The proposal in the first hypothesis appears to be upheld: the age of the worker is directly related to the accident: the older the worker, the greater the severity of the accident.

#### 3.1.2. Hypothesis 2: Age and Number of Days off Work due to the Injury

**Figure 4:**
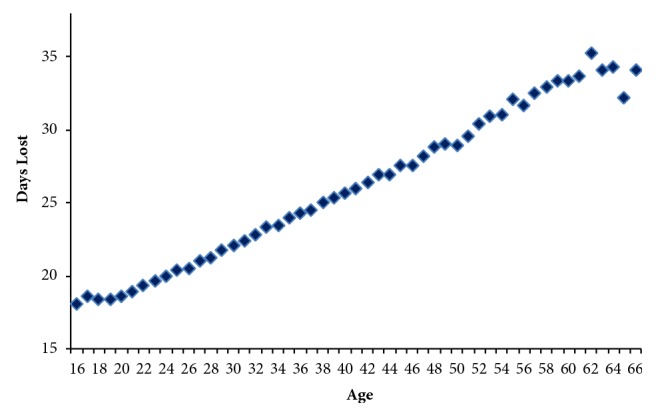
Days lost due to accidents. Spain 1996-2015.* Source.* Prepared by the authors using the anonymous data on occupational accidents provided by the Spanish Subdirección General de Estadística of the Ministerio de Empleo y Seguridad Social.

In Figure 4, it may be seen that the days off work due to the injury increased at a constant rate with age, only interrupted over recent years. The results shown in Table 2 were obtained for the single-factor ANOVA of the average number of days lost due to the injury at each specific age from 16 to 66 years (a total of 50 degrees of freedom).

Determining the average number of days off work due to the injury by age bands, it is seen in Table 3, that, in effect, significant differences occur between the average number of days off work in the different age bands. It may be seen that the average number of days off work increases in each age band, passing from an average number of 19.12 days off work among the youngest workers to 32.91 days off work following accidents involving the oldest workers.

In the same table, it is seen that the average number of days off work due to the injury also increases with age for severe and fatal accidents. We therefore concluded that the average number of days lost following accidents involving young workers, regardless of their severity, was significantly lower than those registered among older workers, as proposed in our Hypothesis 2.

We saw that the average age of workers involved in minor accidents was significantly lower than the average age of workers involved in severe or fatal accidents. We also confirmed significant differences between the average number of days lost following minor accidents and days lost following severe and fatal accidents; it was demonstrated that fewer days were lost following accidents involving younger workers than days lost following accidents involving older workers.

### 3.2. Age and Accumulation of Fatigue

In the following section, we seek to test Hypothesis 3 and Hypothesis 4 on the influence of accumulated fatigue, during both the working day and the working week, on accident rates. In other words, we wish to know whether the accumulation of fatigue will affect all workers equally, regardless of age.

To do so, we verified the values of the following variables: severity, days off work due to injury, and age of the worker in relation to accidents in the morning hours and compared them with the values of the same variables in the afternoon hours. Finally, we compared the values of the same variables in relation to accidents on different days of the week.

#### 3.2.1. Hypothesis 3: Morning and Afternoon Hours

In all, 3,088,376 accidents were selected for this study, which occurred in what we consider a normal working day: between 08:00 in the morning to 19:00 in the afternoon. In Spain, the majority of construction firms start their work between 08:00 and 09:00 hours. They stop for lunch between 13:00 and 14:00 hours in the afternoon and return to work between 15:00 and 16:00 hours in the afternoon. The working day ends, in general, between 18:00 and 19:00 hours. In all, 349,769 accidents were therefore eliminated, which occurred at 14:00 hours, when the majority of construction workers are resting for lunch, and those that occurred between 20:00 hours and 07:00 hours. The aim is to exclude high-risk situations caused by night shifts or excessive working hours (overtime). Likewise, the accidents were classified into morning hours (from 08:00 to 13:00 hours) and afternoon hours (from 15:00 to 19:00 hours).

Practically speaking, we consider that the same construction workers will work on both the morning and the afternoon shifts and that the activities that they perform are neither dependent on the morning nor the afternoon shift. In consequence, if no consideration is given to the accumulated fatigue at work, we understand that there should be no significant differences in the average age of the workers injured in accidents in both the morning and afternoon, and neither should there be differences in the days off work due to the injury, nor in the severity of the accident.

We can see from Table 4 that the number of accidents in the morning hours was always very much higher than those notified in the afternoon hours, but the afternoon accidents involved more days off work. In addition, the percentage of severe and fatal accidents in the afternoon (1.89%) was also higher than in the morning (1.30%), presenting significant differences (t: -36.159; CL 95% of the difference: lower -0.62%, upper -0.56%)

In addition, this higher percentage of severe and fatal accidents was registered in all the years under analysis. Thus, the highest percentages, both in the morning and in the afternoon hours, were registered in 1996 (morning: 1.67%; afternoon: 2.44%) and the lowest in 2009 (morning: 1.06%; afternoon: 1.48%).

The average age of workers involved in accidents in the morning hours (35.26 years) was also lower than the average age of workers involved in accidents in the afternoon hours (35.54) and that difference was also significant (t: -19.910; CL 95% of the difference: lower -0.309, upper -0.254).

Also, as mentioned, there were a higher average number of days off work due to the injury following the accident when it occurred in the afternoon (25.01 days) than when it took place in the morning (23.23 days) being the difference statistically significant (t: -40.149; CL 95% of the difference: lower -1.858, upper -1.685).

It therefore appears to be confirmed that the accidents in the afternoon were more severe and involved more days off work than those that occurred in the morning. Below, we can appreciate the significance of those differences for all age bands.

Analyzing Table 5, we can see that both the accident rates in the morning and in the afternoon include increasingly high percentages of severe accidents as the age of the injured worker increases, as in previous sections. It demonstrates that the age of the worker continues to act as a risk factor in the sector-related accident rates. But, in addition, a further risk factor is observed as the working day advances, perhaps due to accumulated fatigue, which should be taken into account, as it affects all age bands. Thus, the percentages of severe and fatal accidents in the afternoon increased considerably in all the age bands in relation to those registered in the morning hours, although that increase was more intense at older ages of the injured worker.

Our analysis confirms the suspicion that as the working day advances, possibly due to accumulated fatigue, the accident rates for all workers in the sector are affected, from the youngest to the oldest. However, older workers are affected with greater intensity, which supports the proposal in Hypothesis 3.

#### 3.2.2. Hypothesis 4: Day of the Week

We can see a situation in Table 6 that we find difficult to explain: there are fewer accidents as the week progresses. In fact, this pattern is evident throughout the 20 years under analysis. Thus, the highest number of accidents was registered on Monday (25.1%), followed by Tuesday (20.5%), Wednesday (19.2%), Thursday (17.0%), and Friday (15.5%). Subsequently, as is logical, there is Saturday (2.1%) and Sunday (0.6%).

We consider that the construction workers on different days of the week hardly vary and that their activities are not dependent on any one day of the week. In consequence, if accumulated fatigue is not considered, we understand that there should be no significant differences between the average age of the workers involved in accidents on Mondays, on Tuesdays, and on any other day of the week.

We can see from Table 7 that the average age of the worker was older and the average number of days off work due to the injury was higher in relation to accidents on Thursdays and Fridays than in relation to accidents registered on Mondays and Tuesdays.

With regard to accident severity, we can see from Table 7 that fewer severe and fatal accidents occurred as the week progressed; however, the percentage of severe and fatal accidents followed a contrary process. For every one hundred accidents that occurred on Monday, 1.32 were severe or fatal; 1.46 on Tuesday; 1.54 on Wednesday; 1.68 on Thursday; and 1.69 on Friday. The difference between Monday and Friday presented the following confidence intervals: t: -17.162; CL 95% of the difference: lower -0.411%, upper- 0.327%.

Looking at these curious aspects in greater depth, accident rates on Monday and Friday are analyzed below, as the days that represent the highest and the lowest number of accidents in the ordinary working week.

As we can see from Table 7, the average number of days off work on Mondays stood at 22.83 days, while the average number of days off work following accidents on Fridays was 26.18 days, with the following confidence intervals for the difference of means (t: -54.771; CL 95% of the difference: lower -3.471, upper -3.231). In addition, the higher number of days off work on Mondays was registered in 2014 (31.84) and the lowest value in 2004 (20.88). In contrast, Fridays registered the highest number of days off work in 2015 (36.43) and the lowest in 2000 (23.88).

Likewise, the average age of workers involved in accidents on Monday stood at 35.11 years in age and the average age of workers injured on Fridays was 36.07 years (t: -48.865; CL 95% of the difference: lower -0.998, upper -0.921). In addition, the highest value was registered in 2014 (40.62 years old) and the lowest value in 2001 (34.08). The highest value in 2015 (41.27) was, specifically, registered on Fridays and the lowest value in 2000 (34.98).

Analyzing Table 8, we can see from the accident rates on both Mondays and Fridays that the percentages of severe accidents increased with the age of injured worker. As has been confirmed in preceding sections, that observation demonstrates that workers of older age presented a risk factor in relation to accident severity. But, it was also observed that a further risk factor, to take into account in all age bands, was the progression of working days in the week, possibly due to accumulated fatigue. Thus, the percentages of severe accidents on Fridays increased considerably in all age bands in relation to those registered on Mondays.

Our suspicion that as the week progresses, it has an effect on all workers was confirmed, although the differences were not significant at a confidence level of 95% among workers of an older age.

## 4. Conclusions

The average age of the workers involved in severe and fatal accidents in all the years under analysis has been shown to be higher than those accidents registered by workers involved in minor accidents. This difference was statistically significant with an average value of 3.2 years. In consequence, it appears to confirm that the age of the worker injured in an accident is directly related to the severity of the accident: the older the age, the greater the severity.

It has also been confirmed that the average number of days off work following accidents involving young workers was significantly lower than the number of days off work registered for accidents involving older workers. We should attempt to reduce the load of injuries among construction workers, recognizing the vulnerable populations, such as older workers [3]. Likewise, Blanch et al. [25] related the higher number of days lost among older workers with the inherent reduction in corporal elasticity which occurs when aging and with the fact that the injuries could be more severe among older workers.

This information should be taken into account by the governments of those countries that are “aging” with a view to avoiding increases in accident severity and days lost which we know will continue to increase, year on year, as the age of the workers increase.

Moreover, many authors have sought to relate fatigue and accident rates in the construction sector. Thus, the relation between working hours and safety were examined, to show that the factors relating to fatigue had an adverse effect on worker safety [26]. The dangers of fatigue, studies by Folkard and Tucker [27], indicated that human error as a result of fatigue is the most dangerous. In construction, as stated in the Introduction [12], a critical level of fatigue has been calculated, at which point its influence on safety practices starts to be significant, and a linear relation has even been established between levels of fatigue and the error rate.

In this study, our suspicion that the progression of the working day and perhaps the accumulation of fatigue would somehow affect the accident rates of all workers in the sector, from the youngest to the oldest, has been confirmed. However, it has been shown that the most affected were the oldest workers.

The “Monday effect” was defined by Card and McCall [28] and has been confirmed, in so far as if the workers were covered by health insurance, then their accidents were more numerous on Mondays. The demonstration of the existence of moral risk in the accidents that occurred on this day has been shown through graphic images of back injuries with burns and cuts. The first, which can be notified even after one or two days, registered a significant increase on Mondays. The second, in contrast, registered the highest number on Thursdays.

However, a further risk factor to take into account is the progression of the week, possibly due to an accumulation of fatigue, as it affects all age bands. Thus, the percentages of severe accidents on Fridays increased considerably in all the age bands in relation to those registered on Mondays.

This analysis confirms the suspicion that the progression of the week affects all workers, although the differences were not significant to 95% among the older workers. Continuous training activities should be programmed on risk-prevention topics, and perhaps most important of all, there should be a specific intervention in the redesign of jobs, above all in physically and psychologically demanding contexts [25].

This analysis of accidents should be complemented by other similar studies in other countries. In addition, risk-prevention measures should be analyzed among older workers which will permit reductions in the incidence of accident severity in this group of workers. We consider that these studies should be of utility to risk-prevention managers organizing on-site risk prevention in their fight against the high levels of accident rates notified in the construction sector.

### 4.1. Impact on the Industry

In this study, it has been confirmed that the severity and the duration of the accident-related sick leave are directly related to age, in jobs with significant physical (efforts) requirements. This information should be considered by the governments of those countries that have “aging” populations, with the objective of preventing any further increase in accident severity and days off work due to the injury following accidents, which as we know will rise year by year as the workers grow older.

This study also confirms that as the working day and week progress, perhaps due to the accumulated fatigue effect that is expended, it affects the accident rates in the sector among all workers, although with greater intensity among older workers; we consider that training courses should be programmed in accident prevention and that the jobs should be redesigned in accordance with the age of the worker, above all in demanding physical contexts. We also consider it convenient and very necessary to raise the awareness of entrepreneurs and risk-prevention managers; it will become essential in the near future to train construction workers of all ages (especially older workers), in ergonomic aspects, with the objective of reducing injuries that are a consequence of posture-related and physical effort that lead to an accumulation of fatigue that are, in turn, a cause of higher accident rates.

## Figures and Tables

**Table 1:** Number of construction accidents by severity, age, and average number of days off work. Spain 1996-2015.

	Number of Total Accidents (1)	Rate*∗* (2)	Average Age [years] (3)	Average Number of Days Off Work (4)	Number of Severe+Fatal Accidents (5)	% Severe+Fatal (6)
1996	134,144	15.9	35.27	25.51	2,645	1.97%
1997	147,103	16.4	35.12	23.28	2,656	1.81%
1998	179,295	17.5	34.71	22.50	3,048	1.70%
1999	224,868	18.8	34.44	23.15	3,379	1.50%
2000	248,727	18.8	34.31	22.08	3,519	1.41%
2001	260,765	18.3	34.28	22.60	3,880	1.49%
2002	259,522	17.3	34.40	22.64	3,959	1.53%
2003	238,808	15.3	34.68	23.70	4,353	1.82%
2004	235,430	13.9	34.75	22.17	4,096	1.74%
2005	250,372	13.5	34.45	22.79	3,789	1.51%
2006	262,225	12.7	34.68	22.42	3,734	1.42%
2007	263,490	12.4	35.04	23.71	3,672	1.39%
2008	193,769	10.7	35.85	22.10	2,632	1.36%
2009	149,738	9.0	36.95	26.90	1,859	1.24%
2010	105,817	8.5	37.73	29.12	1,487	1.41%
2011	84,674	7.7	38.54	30.23	1,243	1.47%
2012	55,392	6.3	39.31	31.12	874	1.58%
2013	45,517	6.0	39.89	33.10	640	1.41%
2014	46,294	6.3	40.38	32.96	703	1.52%
2015	52,195	6.8	40.73	33.94	749	1.44%

Total	3,438,145		35.31	24.01	52,917	1.54%

*Notes*. *∗*Accident rate for every 100 workers, http://www.empleo.gob.es/es/estadisticas/monograficas_anuales/EAT/2015/index.htm. *Source*. Prepared by the authors using the anonymous data on occupational accidents provided by the Subdirección General de Estadística of the Ministerio de Empleo y Seguridad Social.

**Table 2:** Results obtained for the single-factor ANOVA of days off work due to the injury and age. Spain 1996-2015.

ANOVA	Sum of squares	d.f.	Quadratic measure	F	Sig.
Between groups	57,517,893.282	50	1,150,357.866	979.333	0.000
Within groups	4,024,327,883.864	3,426,026	1,174.634		

**Table 3:** Accidents by ages and by days off work. Spain 1996-2015.

Age	Accidents		CL for the average at 95%		Accidents		CL for the average at 95%	
	Totals	Average days lost	Lower Limit [days]	Upper Limit [days]	S+F	Average days lost	Lower Limit [days]	Upper Limit [days]
16-24	683,446	19.12	19.06	19.18	7,511	90.33	88.45	92.20
25-34	1,142,450	21.85	21.79	21.9	14,334	97.84	96.34	99.33
35-44	859,460	25.36	25.28	25.43	14,121	108.08	106.48	109.69
45-54	528,429	29.02	28.91	29.13	11,223	111.07	109.22	112.91
>54	224,360	32.91	32.72	33.11	5,728	112.73	110.08	115.37

Total	3,438,145	24.01	23.97	24.04	52,917	103.92	103.11	104.73

*Notes.* All differences analyzed present a p-value <0.01. *Source.* Prepared by the authors using the anonymous data on occupational accidents provided by the Subdirección General de Estadística of the Ministerio de Empleo y Seguridad Social.

**Table 4:** Number of accidents due to severity and morning and afternoon hours. Spain 1996-2015.

	Morning Hours				Afternoon Hours				Totals
	Minor	S + F	Age [Years]	Days Lost	Minor	S + F	Age [Years]	Days Lost	
1996	86,233	1,466	35.2	24.7	35,692	892	35.5	26.6	124,283
1997	94,659	1,453	35.1	22.6	39,556	893	35.3	24.2	136,561
1998	116,885	1,650	34.7	21.9	47,261	1,020	35	23.1	166,816
1999	148,405	1,923	34.4	22.7	57,199	1,065	34.6	23.6	208,592
2000	163,120	1,956	34.3	21.4	63,555	1,146	34.6	22.9	229,777
2001	169,808	2,157	34.3	21.9	67,080	1,273	34.5	23.5	240,318
2002	166,221	2,174	34.4	22	69,551	1,295	34.5	23.4	239,241
2003	150,222	2,340	34.7	23	64,816	1,448	35	24.6	218,826
2004	143,246	2,125	34.7	21.2	61,388	1,338	35.1	23.4	208,097
2005	158,655	1,994	34.4	21.8	64,899	1,307	34.8	24.1	226,855
2006	166,548	2,016	34.6	21.6	68,238	1,243	35	23.5	238,045
2007	156,563	1,929	35	22.9	61,768	1,044	35.3	24.9	221,304
2008	110,142	1,246	35.8	21.5	43,666	741	36.1	23.2	155,795
2009	87,936	938	37	26	36,372	546	37.1	28.3	125,792
2010	65,623	808	37.8	28.1	27,699	456	37.9	30.3	94,586
2011	52,933	665	38.6	29.2	22,118	402	38.7	31.2	76,118
2012	34,456	494	39.4	30.3	14,325	270	39.5	32.4	49,545
2013	28,265	368	39.9	32.1	11,576	195	40	35	40,404
2014	28,740	406	40.3	32.1	11,724	208	40.6	34.2	41,078
2015	32,387	428	40.7	33.4	13,303	225	40.9	34.6	46,343

Total	2,161,047	28,536	35.3	23.2	881,786	17,007	35.5	25	3,088,376

*Source.* Prepared by the authors using the anonymous data on occupational accidents provided by the Subdirección General de Estadística of the Ministerio de Empleo y Seguridad Social.

**Table 5:** Percentages of severe and fatal (S+F). Differences by age. Spain 1996-2015.

Age [Years]	% S+F		Difference	CL 95% of the difference		P-value
	Morning	Afternoon		Lower	Upper	
16-24	0.92%	1.32%	-0.41%	-0.35%	-0.47%	<0.01
25-34	1.06%	1.53%	-0.47%	-0.42%	-0.52%	<0.01
35-44	1.38%	2.07%	-0.69%	-0.63%	-0.76%	<0.01
45-54	1.82%	2.58%	-0.76%	-0.67%	-0.86%	<0.01
>54	2.24%	3.00%	-0.76%	-0.61%	-0.92%	<0.01

*Source.* Prepared by the authors using the anonymous data on occupational accidents provided by the Subdirección General de Estadística of the Ministerio de Empleo y Seguridad Social.

**Table 6:** Number of construction sector-related accidents. Totals by day of the week. Spain 1996-2015.

	Monday	Tuesday	Wednesday	Thursday	Friday	Saturday	Sunday	Total
1996	32,620	27,168	25,285	23,055	21,531	3,494	991	134,144
1997	35,419	30,210	27,664	25,056	23,928	3,671	1,155	147,103
1998	43,122	35,655	34,758	31,268	29,001	4,359	1,132	179,295
1999	53,731	45,905	42,420	39,397	36,290	5,701	1,424	224,868
2000	61,674	49,681	47,244	42,307	39,385	6,554	1,882	248,727
2001	65,206	52,772	50,081	44,104	40,408	6,265	1,929	260,765
2002	66,543	52,602	49,441	43,838	39,477	5,951	1,670	259,522
2003	60,430	50,168	45,140	40,616	36,314	4,912	1,228	238,808
2004	59,594	48,218	45,672	40,717	36,112	4,273	844	235,430
2005	63,308	52,417	48,898	42,102	38,098	4,635	914	250,372
2006	67,086	55,068	50,570	44,309	38,662	4,982	996	261,673
2007	68,877	54,362	50,698	44,244	39,541	4,816	952	263,490
2008	49,783	40,865	37,566	31,830	29,581	3,369	775	193,769
2009	37,845	30,423	29,376	25,429	23,191	2,695	779	149,738
2010	26,544	21,742	20,182	18,457	16,358	1,934	600	105,817
2011	21,059	17,293	16,447	14,309	13,505	1,600	461	84,674
2012	14,195	11,154	10,521	9,257	8,800	1,103	362	55,392
2013	11,367	9,141	8,528	7,819	7,292	995	375	45,517
2014	11,219	9,414	8,777	7,859	7,407	1,228	390	46,294
2015	12,705	10,508	10,008	8,925	8,370	1,295	384	52,195

Totals	862,327	704,766	659,276	584,898	533,251	73,832	19,243	3,437,593∗

*Notes*. *∗*No data due to lack of information: 592. *Source.* Prepared by the authors using the anonymous data on occupational accidents provided by the Subdirección General de Estadística of the Ministerio de Empleo y Seguridad Social.

**Table 7:** Construction accidents: Days lost, age, and severity, by day of the week. Spain 1996-2015.

	Total Accidents	Days Lost	CL for average at 95%		Age [Years]	CL for average at 95%		S + F Accidents
			Lower	Upper		Lower	Upper	
Monday	862,327	22.83	22.76	22.90	35.11	35.09	35.13	11,377
Tuesday	704,766	22.99	22.91	23.07	35.04	35.01	35.07	10,270
Wednesday	659,276	23.49	23.40	23.57	35.15	35.12	35.18	10,157
Thursday	584,898	24.85	24.76	24.94	35.38	35.35	35.40	9,848
Friday	533,251	26.18	26.08	26.28	36.07	36.04	36.10	9,003
Saturday	73,832	28.47	28.19	28.74	35.39	35.31	35.47	1,859
Sunday	19,243	28.96	28.43	29.49	36.58	36.43	36.74	396

Total	3,437,593	24.01	23.97	24.04	35.31	35.30	35.32	52,910

*Notes.* p<0.01. *Source.* Prepared by the authors using the anonymous data on occupational accidents provided by the Subdirección General de Estadística of the Ministerio de Empleo y Seguridad Social.

**Table 8:** Mondays and Fridays: severity by age. Spain 1996-2015.

Age [Years]	% S+F		Difference	CL 95% of the difference		P-value
	Mondays	Fridays		Lower	Upper	
16-24	0.94%	1.30%	-0.36%	-0.28%	-0.44%	<0.01
25-34	1.07%	1.42%	-0.35%	-0.33%	-0.41%	<0.01
35-44	1.37%	1.76%	-0.39%	-0.30%	-0.48%	<0.01
45-54	1.85%	2.16%	-0.31%	-0.19%	-0.43%	<0.01
>54	2.35%	2.49%	-0.14%	-0.33%	-0.41%	>0.05

*Source.* Prepared by the authors using the anonymous data on occupational accidents provided by the Subdirección General de Estadística of the Ministerio de Empleo y Seguridad Social.

## Data Availability

The data used to support the findings of this study are available from the General Subdirectorate of Statistics [Subdirección General de Estadísticas] of the Ministerio de Empleo y Seguridad Social of Spain.

## References

[1] Anaya C., Ayuso J. L., Friestersch E. (2015). *Safer and healthier work at any age. Country Inventory: Spain*.

[2] Healthy Workplaces for All Ages. https://healthy-workplaces.eu/previous/all-ages-2016/en/campaign-materials/guide.

[3] Schwatka N. V., Butler L. M., Rosecrance J. R. (2012). An aging workforce and injury in the construction industry. *Epidemiologic Reviews*.

[4] Salminen S. (2004). Have young workers more injuries than older ones? An international literature review. *Journal of Safety Research*.

[5] Yeoh H. T., Lockhart T. E., Wu X. (2013). Non-fatal occupational falls on the same level. *Ergonomics*.

[6] Mazina D., Donneau A.-F., Mairiaux P. H. (2012). Determinants of sickness absence duration after an occupational back injury in the belgian population. *American Journal of Industrial Medicine*.

[7] Huang X., Hinze J. (2003). Analysis of construction worker fall accidents. *Journal of Construction Engineering and Management*.

[8] Dong X. S., Fujimoto A., Ringen K., Men Y. (2009). Fatal falls among Hispanic construction workers. *Accident Analysis & Prevention*.

[9] Chau N., Gauchard G. C., Siegfried C. (2004). Relationships of job, age, and life conditions with the causes and severity of occupational injuries in construction workers. *International Archives of Occupational and Environmental Health*.

[10] Hinze J., Devenport J. N., Giang G. (2006). Analysis of construction worker injuries that do not result in lost time. *Journal of Construction Engineering and Management*.

[11] Layne L. A., Pollack K. M. (2004). Nonfatal occupational injuries from slips, trips, and falls among older workers treated in hospital emergency departments, United States 1998. *American Journal of Industrial Medicine*.

[12] Fang D., Jiang Z., Zhang M., Wang H. (2015). An experimental method to study the effect of fatigue on construction workers' safety performance. *Safety Science*.

[13] Horwitz I. B., McCall B. P. (2004). Disabling and fatal occupational claim rates, risks, and costs in the Oregon construction industry 1990-1997. *Journal of Occupational and Environmental Hygiene*.

[14] Goldenhar L. M., Hecker S., Moir S., Rosecrance J. (2003). The “goldilocks model” of overtime in construction: Not too much, not too little, but just right. *Journal of Safety Research*.

[15] Cattledge G. H., Hendricks S., Stanevich R. (1996). Fatal occupational falls in the U.S. construction industry, 1980-1989. *Accident Analysis & Prevention*.

[16] Cattledge G. H., Schneiderman A., Stanevich R., Hendricks S., Greenwood J. (1996). Nonfatal occupational fall injuries in the West Virginia construction industry. *Accident Analysis & Prevention*.

[17] Hinze J., Huang X., Terry L. (2005). The nature of struck-by accidents. *Journal of Construction Engineering and Management*.

[18] Hinze J., Bren D. (1996). Analysis of fatalities and injuries due to powerline contacts. *Journal of Construction Engineering and Management*.

[19] Macedo A. C., Silva I. L. (2005). Analysis of occupational accidents in Portugal between 1992 and 2001. *Safety Science*.

[20] Wigglesworth E. (2006). Occupational injuries by hour of day and day of week: A 20-year study. *Australian and New Zealand Journal of Public Health*.

[21] Kines P. (2002). Construction workers' falls through roofs: fatal versus serious injuries.. *Journal of Safety Research*.

[22] Real Decreto 625/2014 of the Ministry of Employment and Social Security, *Boletín Oficial del Estado*, 2014, https://www.boe.es/boe/dias/2014/07/21/pdfs/BOE-A-2014-7684.pdf

[23] Huete L. (2007). La actuación del Ministerio Fiscal en siniestralidad laboral: una guía práctica. *Prosecutor's Office of Provincial Court of Ciudad Real*.

[24] Control de la aplicación de las Normas Internacionales del Trabajo para España-Comentarios de la Comisión de Expertos (CEACR)-Observación sobre la aplicación del Convenio C155-Artículo 9: Sanciones. http://www.ilo.org/dyn/normlex/es/f?p=1000:13100:0::NO::P13100_COMMENT_ID,P13100_LANG_CODE:2268123,en:NO.

[25] Blanch A., Torrelles B., Aluja A., Salinas J. A. (2009). Age and lost working days as a result of an occupational accident: A study in a shiftwork rotation system. *Safety Science*.

[26] Dong X. (2005). Long workhours, work scheduling and work-related injuries among construction workers in the United States. *Scandinavian Journal of Work, Environment & Health*.

[27] Folkard S., Tucker P. (2003). Shift work, safety and productivity. *Occupational Medicine *.

[28] Card D., McCall B. P. (1996). Is workers' compensation covering uninsured medical costs? Evidence from the “monday effect”. *Industrial and Labor Relations Review*.

